# Altersspezifisches Melanomrisiko im Zusammenhang mit melanozytären Nävi: Eine gepoolte Analyse aus dem M‐SKIP‐Projekt

**DOI:** 10.1111/ddg.15992_g

**Published:** 2026-09-08

**Authors:** Giulia Doi, Aurora Gaeta, Simone Ribero, Nelleke Gruis, Julia Newton‐Bishop, David Polsky, DeAnn Lazovich, Paola Ghiorzo, Gloria Ribas, Chiara Menin, Alexander J. Stratigos, Gabriella Guida, Susana Puig, Maria Concetta Fargnoli, Peter A. Kanetsky, Paola Queirolo, Vincenzo Bagnardi, Veronique Bataille, Sara Raimondi, Sara Gandini, M‐SKIP study group

**Affiliations:** ^1^ Molecular and Pharmaco‐Epidemiology Unit Department of Experimental Oncology IEO European Institute of Oncology IRCCS Milan Italy; ^2^ Department of Statistics and Quantitative Methods University of Milan‐Bicocca, Italy; ^3^ Department of Medical Sciences Dermatologic Clinic University of Turin Turin Italy; ^4^ Department of Dermatology Leiden University Medical Center Leiden The Netherlands; ^5^ Institute of Medical Research at St James's University of Leeds Leeds UK; ^6^ Ronald O. Perelman Department of Dermatology New York University Grossman School of Medicine NYU Langone Health New York USA; ^7^ Division of Epidemiology and Community Health University of Minnesota Minneapolis Minnesota USA; ^8^ Department of Internal Medicine and Medical Specialties University of Genoa Genoa Italy; ^9^ Cancer Genetics IRCCS Ospedale Policlinico San Martino Genoa Italy; ^10^ Dpdt. Oncologia medica y hematologia Fundación Investigación Clínico de Valencia Instituto de Investigación Sanitaria‐ INCLIVA Valencia Spain; ^11^ Diagnostic Immunology and Molecular Oncology Unit Veneto Institute of Oncology IOV‐IRCCS Padua Italy; ^12^ 1st Clinic of Dermatological and Venereal Diseases Medical School National and Kapodistrian University of Athens Andreas Sygros Hospital Athina Greece; ^13^ Department of Translational Biomedicine and Neuroscience (DiBraiN) University of Bari “A. Moro” Bari Italy; ^14^ Melanoma Unit Dermatology Department Hospital Clinic Barcelona Universitat de Barcelona Institut d'Investigacions Biomèdiques August Pi I Sunyer (IDIBAPS), Spain; ^15^ CIBER de Enfermedades Raras Barcelona Spain; ^16^ San Gallicano Dermatological Institute – IRCCS Rome Italy; ^17^ Department of Cancer Epidemiology H. Lee Moffitt Cancer Center and Research Institute Tampa Florida USA; ^18^ Melanoma Sarcoma and Rare Tumors Oncology Department IEO European Institute of Oncology IRCCS Milan Italy; ^19^ Twin Unit and genetic epidemiology department King's College London London UK; ^20^ Current Affiliation: Recordati SpA Milan Italy

**Keywords:** Alter, Melanom, MC1R, Nävi, primäre und sekundäre Prävention, Age, melanoma, MC1R, nevi, primary and secondary prevention

## Abstract

**Zielsetzung:**

Diese Studie untersucht den Zusammenhang zwischen dem Melanomrisiko und der Gesamtanzahl der Nävi am Körper sowie dem Vorliegen atypischer Nävi bei jüngeren (< 40 Jahre) und älteren (> 60 Jahre) Personen.

**Methoden:**

Im Rahmen des M‐SKIP‐Projekts wurde eine gepoolte Analyse auf der Grundlage mehrerer Fall‐Kontroll‐Studien zu Melanomen durchgeführt. Die Zusammenhänge wurden anhand studienspezifischer, um potenzielle Störfaktoren bereinigter *Odds Ratios* (ORs) bewertet.

**Ergebnisse:**

Es wurden neun Fall‐Kontroll‐Studien einbezogen, in denen gewöhnliche Nävi (2.674 Melanomfälle/2.343 Kontrollen) und atypische Nävi (2.459 Fälle/1.740 Kontrollen) analysiert wurden. Eine hohe Anzahl gewöhnlicher Nävi war sowohl bei jüngeren (zusammengefasste [s]OR = 2,56; 95 % CI 1,64–4,01; I^2^ = 31 %) als auch bei älteren Personen (sOR = 2,06; 95 % CI 1,06–4,00; I^2^ = 67 %) mit einem erhöhten Melanomrisiko assoziiert, wobei kein signifikanter Unterschied zwischen den Altersgruppen festgestellt wurde (p = 0,57). Im Gegensatz dazu hatten jüngere Personen ein deutlich höheres Melanomrisiko beim Vorliegen von mindestens einem atypischen Nävus (sOR = 4,84; 95 % CI 2,18–10,76; I^2^ = 65 %) im Vergleich zu älteren Personen (sOR = 1,71; 95 % CI 1,07–2,74; I^2^ = 39 %), mit einem p‐Wert von 0,02 für den Unterschied.

**Schlussfolgerungen:**

Diese Ergebnisse deuten auf altersspezifische Unterschiede beim Melanomrisiko in Verbindung mit der Nävuszahl hin. Insbesondere atypische Nävi bedeuten bei jüngeren Menschen ein höheres Risiko. Dies könnte erhebliche Auswirkungen auf Präventionsstrategien und die klinische Behandlung haben, insbesondere im Hinblick auf bessere Identifizierung von Hochrisikogruppen für gezielte Sekundärprävention.

## EINLEITUNG

Das Melanom, das durch das unkontrollierte Wachstum von Melanozyten gekennzeichnet ist, ist eine der bösartigsten Formen von Hautkrebs^1^ und zählt zu den häufigsten Krebsarten bei jüngeren Menschen.^2^ Obwohl es seltener auftritt als andere Hautkrebsarten, steigt seine Inzidenz weltweit an, was zu ernsthaften Bedenken hinsichtlich der öffentlichen Gesundheit führt.^3^ Das Melanomrisiko steigt mit fortschreitendem Alter, jedoch variiert die Inzidenz je nach Alter und Geschlecht. Während Männer im Allgemeinen häufiger an Melanomen erkranken und ein höheres Mortalitätsrisiko haben, ist die Wahrscheinlichkeit, dass Frauen unter 50 Jahren mit dieser Krankheit diagnostiziert werden, fast doppelt so hoch wie bei Männern.^4^ Das Verständnis dieser demografischen Muster ist wichtig für die Entwicklung gezielter Präventions‐ und Behandlungsstrategien.

Das Risiko, an einem kutanen Melanom zu erkranken, steigt mit der Zahl gewöhnlicher melanozytärer Nävi.^3^ Die Gesamtanzahl der Nävi am Körper (TBNC) ist einer der stärksten Risikofaktoren für Melanome, mit einem viel höheren relativen Risiko als Umweltfaktoren.^5^ Sonnenlicht mag zwar an der Nävogenese beteiligt sein, doch, wie Familien‐ und Zwillingsstudien zeigen, unterliegen Nävi größtenteils der genetischen Kontrolle.^6^ Querschnittsstudien haben bislang eine geringere Prävalenz von Nävi bei älteren Menschen gezeigt, doch sie befassten sich nicht mit dem natürlichen Nävus‐Verlauf, der aus ihren Ergebnissen nur vage abgeleitet werden kann.^7^, ^8^, ^9^ Ein besseres Verständnis der Biologie von Melanomen und Nävi könnte dabei helfen, sekundäre Präventionsstrategien anzupassen.

Auf der Grundlage früherer Querschnittsstudien wurde spekuliert, dass Nävi bei kaukasischen Populationen typischerweise nach der vierten Lebensdekade auftreten und bei älteren Menschen seltener sind. Personen, die anfällig für Melanome sind, weisen hingegen häufig eine größere Anzahl gewöhnlicher und atypischer Nävi auf, die bis ins mittlere Alter oder darüber hinaus bestehen bleiben.^3^ Die meisten Fall‐Kontroll‐Studien liefern nur Querschnittsdaten, sodass es nicht einfach ist, das Verhalten von Nävi über die gesamte Lebensdauer hinweg genau vorherzusagen. Die meisten Längsschnittstudien wurden an kleinen Hochrisikogruppen durchgeführt, wie beispielsweise Patienten mit Melanomen, solchen mit Melanomen in der Familienanamnese oder Patienten mit atypischen Nävi,^10^, ^11^, ^12^ und die Ergebnisse lassen sich kaum auf die allgemeine Bevölkerung übertragen. Nur wenige Längsschnittstudien haben untersucht, ob die Nävuszahl mit zunehmendem Alter abnimmt, und es wurden widersprüchliche Ergebnisse berichtet.^10^, ^11^, ^12^

In dieser Studie wollten wir den Zusammenhang zwischen Nävi und Melanomen in Abhängigkeit vom Alter anhand einer gepoolten Analyse einer großen Stichprobe untersuchen.

## MATERIAL UND METHODEN

### Daten

Die Daten stammen aus dem Melanocortin‐1 Receptor, Skin Cancer and Phenotypic Characteristics (M‐SKIP‐) Projekt, einer internationalen Partnerschaft zum Melanocortin‐1‐Rezeptor‐ (MC1R‐) Gen, zum Hautkrebsrisiko und zu phänotypischen Merkmalen.^13^ Die Autoren von M‐SKIP lieferten Informationen zum jeweiligen Herkunftsland, zum Studiendesign, zum Ursprung der Kontrollen, zur Anwendung des Fall‐Kontroll‐Abgleichs, zu Phänotypisierungs‐ und Genotypisierungs‐Methoden sowie zur Art der Blindheit. Variablen wie Alter, Geschlecht, ethnische Zugehörigkeit, BMI, Raucherstatus, Sonnenexposition (intermittierend und kontinuierlich/chronisch), Hautkrebs in der Familienanamnese, Lokalisation des Melanoms, Histologie des Melanoms, Breslow‐Tiefe, Haar‐ und Augenfarbe, Hauttyp, Epheliden, Lentigines solares und Anzahl gewöhnlicher und atypischer Nävi standen zur Verfügung. Der M‐SKIP‐Datensatz umfasste 39 Forschungsstudien, es wurden aber nur 9 Fall‐Kontroll‐Studien berücksichtigt, die Informationen zu Alter, sporadischem Hautmelanom und der Gesamtanzahl gewöhnlicher und/oder atypischer Nävi am Körper enthielten. Die kontinuierliche Variable, die die Gesamtanzahl der gewöhnlichen Nävi angibt, wurde in Kategorien unterteilt. Wenn diese Gesamtanzahl in Kategorien angegeben wurde, wurde der Median zwischen der oberen und unteren Anzahl der Klasse genommen. Studien mit einer Anzahl gewöhnlicher Nävi gegen Null oder fehlenden Angaben wurden ausgeschlossen. Darüber hinaus wurden Familienstudien oder Studien mit Kontrollpersonen, die ein erhöhtes Hautkrebsrisiko aufweisen, nicht berücksichtigt. Weitere Details sind im Online‐Ergänzungsmaterial aufgeführt.

### Analyse

**ABBILDUNG 1:**
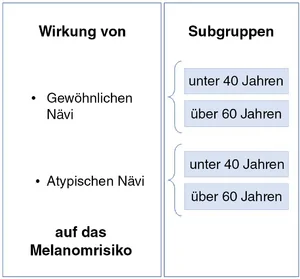
Schema der Subgruppenanalyse.

Es wurden zwei unabhängige Analysen durchgeführt (Abbildung 1): eine zur Auswirkung der Gesamtanzahl gewöhnlicher Nävi auf das Melanomrisiko und eine zweite zum Zusammenhang zwischen mindestens einem atypischen Nävus und dem Melanomrisiko. Gemäß dem *International Agency for Research on Cancer* (IARC‐) Protokoll zur Identifizierung und Erfassung von Nävi in epidemiologischen Studien^14^ ist ein atypischer Nävus definiert als ein Nävus mit einer makulären Komponente in mindestens einem Bereich, das mindestens drei der folgenden Merkmale aufweist: Durchmesser > 5 mm, unscharfe oder verschwommene Ränder, unregelmäßiger Rand, der zu einer ungewöhnlichen Form führt, unterschiedliche Farbtöne (gemäß der Fitzpatrick‐Klassifikation^15^), flache und unebene Komponenten. Angesichts der substanziellen Diversität der Nävuszahl in verschiedenen Altersgruppen haben wir die Stichprobe in drei Subgruppen unterteilt: unter 40 Jahre,^16^ zwischen 40 und 60 Jahre und über 60 Jahre.^17^ Es wurden Analysen durchgeführt, in denen die beiden extremen Altersgruppen (< 40 Jahre vs. > 60 Jahre) verglichen wurden. Die mittlere Altersgruppe (40 ≤ Alter ≤ 60) wurde ausgeschlossen, um eine altersspezifische Wirkung beurteilen zu können.

### Gepoolte Schätzung

Alle erhobenen Daten wurden für eine gepoolte Analyse verwendet.^18^ Die Anzahl der gewöhnlichen Nävi wurde in eine binäre Variable umgewandelt, die in Werte oberhalb und unterhalb der studienspezifischen medianen Anzahl von Nävi in der Kontrollgruppe kategorisiert wurde. Die Variable „atypische Nävi” wurde definiert als das Vorliegen von mindestens einem Nävus vs. das Fehlen desselben. Zur Bewertung des Melanomrisikos in jeder Studie wurde ein logistisches Regressionsmodell angepasst und die Odds Ratios (ORs) mit 95 %‐Konfidenzintervallen (95 % CI) angegeben. Für jede Studie wurde ein multivariates Modell angepasst, das um alle signifikanten Kovariaten bereinigt wurde. Soweit verfügbar, wurden die folgenden Kovariaten einbezogen: gewöhnliche/atypische Nävi (für die beiden unabhängigen Analysen), Geschlecht, intermittierende und kontinuierliche Sonnenexposition, phänotypische Merkmale – wie Haar‐ und Augenfarbe, Hauttyp, Melanom in der Familienanamnese und Varianten im MC1R‐Gen – unter Berücksichtigung des Vorliegens oder Nichtvorliegens mindestens einer Variante des Gens. Alle studienspezifischen ORs wurden zu einer gepoolten Schätzung mit einem Zufallseffekt‐Modell zusammengefasst, das aus einem Durchschnitt der einzelnen Schätzungen besteht, gewichtet nach ihren inversen marginalen Varianzen.^19^ Die Ergebnisse wurden in Walddiagrammen dargestellt.

Die Heterogenität zwischen den Studien wurde mit der I^2^‐Statistik untersucht, die denjenigen Anteil der Gesamtvariabilität zwischen den Studien quantifiziert, der eher auf Heterogenität als auf Zufall zurückzuführen ist. Es wurden Metaregressionen und Subgruppenanalysen durchgeführt, um die Rolle der Merkmale der Studienpopulation und der methodischen Merkmale jeder Studie zu bewerten. Darüber hinaus wurden Metaregressionen verwendet, um Unterschiede zwischen jüngeren und älteren Personen in der Gesamtanalyse zu untersuchen. Es wurden Sensitivitätsanalysen durchgeführt, wobei auch Studien mit Personen mit einem höheren Melanomrisiko, die aufgrund der Einschlusskriterien ausgeschlossen worden waren, wieder in die Analyse einbezogen wurden.

## ERGEBNISSE

Die Merkmale der eingeschlossenen Studien sind in Tabelle 1 aufgeführt: Acht Fall‐Kontroll‐Studien^20^, ^21^, ^22^, ^23^, ^24^, ^25^, ^26^, ^27^, ^28^, ^29^, ^30^, ^31^, ^32^, ^33^, ^34^, ^35^, ^36^, ^37^ wurden in die Analyse der gewöhnlichen Nävi einbezogen, an der 2.674 Personen mit kutanen Melanomen und 2.343 Kontrollen teilnahmen. Die Subgruppe der jüngeren Personen umfasste 605 Melanomfälle und 433 Kontrollen, während der Datensatz der älteren Personen 533 Fälle von kutanen Melanomen und 615 Kontrollen enthielt. Die Studie von Polsky^36^ bezog keine älteren Menschen ein und wurde daher aus der Analyse der älteren Subgruppe ausgeschlossen. Die meisten Studien wurden in Europa, hauptsächlich in Italien, durchgeführt, und für jede Fall‐Kontroll‐Studie wurden die Genotypisierungsanalysen in einem einzigen spezialisierten Zentrum durchgeführt. Die Quelle der Kontrollen variierte von Studie zu Studie. Der Genotyp wurde durch Sequenzierungsanalyse bestimmt, während phänotypische Faktoren und die Nävuszahl durch Untersuchung durch einen Experten oder durch selbst ausgefüllte Fragebögen ermittelt wurden. Subgruppenanalysen zur Untersuchung der Heterogenität zwischen den Studien sind in den Online‐Ergänzungstabellen S1 und S2 dargestellt.

**TABELLE 1:** Merkmale der in die Analyse einbezogenen Studien.

Erstautor	Jahr	Land	Fälle/Kontrollen (N)	Alter (Mittel – min–max)	Gewöhnliche Nävi (Median – IQR)	Ursprung der Kontrollen	Prüfung der Nävuszahl	Beurteilung des Phänotyps	Inklusion in die Analyse gewöhnlicher Nävi	Inklusion in die Analyse atypischer Nävi
Gruis^20^, ^21^, ^22^	2001	Niederlande	114/343	55,49 (24,09–79,79)	14 (5–33)	Krankenhaus	Untersuchung durch einen Experten	Untersuchung durch einen Experten	x	x
Ribas^23^	2007	Spanien	102/155	51,50 (22–87)	12 (12–37)	Bevölkerung	Fragebogen zur Selbstausfüllung oder Untersuchung durch einen Experten	Fragebogen zur Selbstausfüllung oder Untersuchung durch einen Experten	x	
Fargnoli^24^, ^25^, ^26^, ^27^	2008	Italien	155/162	49,34 (17–80)	30 (5–30)	Krankenhaus	Untersuchung durch einen Experten	Untersuchung durch einen Experten	x	x
Bishop^28^	2009	UK	943/485	53,41 (17–86)	28 (13–60)	Krankenhaus	Untersuchung durch einen Experten	Fragebogen zur Selbstausfüllung	x	x
Stratigos^29^, ^30^, ^31^	2009	Griechenland	73/120	46,02 (19–85)	5 (5–30)	Krankenhaus	Untersuchung durch einen Experten	Befragung durch Mediziner oder Untersuchung durch einen Experten (Mediziner)	x	x
Kanetsky^44^, ^45^	2010	USA	713/245	49,25 (19,39–87,76)	/	Bevölkerung	Untersuchung durch einen Experten	Fragebogen zur Selbstausfüllung		x
Ghiorzo^32^, ^33^, ^34^, ^35^	2012	Italien	330/216	54,73 (19‐85)	5 (5–30)	Gesunde Individuen	Untersuchung durch einen Experten für Fälle und Kontrollen	Untersuchung durch einen Experten für Fälle und der Hälfte der Kontrollen (andere: Fragebogen zur Selbstausfüllung)	x	x
Polsky^36^	2014	USA	875/765	46,36 (25–59)	45 (15–45)	Bevölkerung	Fragebogen zur Selbstausfüllung	Fragebogen zur Selbstausfüllung	x	
Guida^37^	2015	Italien	82/97	52,59 (12–89)	25 (25–64)	Krankenhaus	Untersuchung durch einen Experten	Untersuchung durch einen Experten	x	x
***Studien, die aus der Hauptanalyse ausgeschlossen, aber in eine Sensitivitätsanalyse einbezogen wurden***										
Menin^38^	2011	Italien	116/168	42,29 (15,64–82,52)	30 (5–30)	Gesunde Individuen	Untersuchung durch einen Experten bei/für Fälle; Fragebogen zur Selbstausfüllung	Untersuchung durch einen Experten	x	x
Puig^39^, ^40^, ^41^, ^42^, ^43^	2013	Spanien	278/113	51,06 (13,65–93,56)	25 (25–75)	Hochrisiko‐Patienten mit Melanom	Untersuchung durch einen Experten	Fragebogen zur Selbstausfüllung oder Untersuchung durch einen Experten	x	x

Für jede Studie enthält die Tabelle folgende Informationen: Erstautor, Erscheinungsjahr, Land, Zahl der Melanomfälle und Kontrollen, Durchschnittsalter mit Minimal‐ und Maximalwerten, Median und Interquartilbereich (IQR) der gewöhnlichen Nävi, Ursprung der Kontrollen, Methoden zur Bewertung der Nävuszahl und phänotypischen Merkmale sowie Angabe, ob die Studie in die Analysen gewöhnlicher und/oder atypischer Nävi einbezogen wurde.

Die Online‐Ergänzungstabellen S3a (jüngere Gruppe) und S3b (ältere Gruppe) enthalten die rohen und angepassten ORs für jede Studie unter Berücksichtigung einer Reihe signifikanter Störvariablen.

**ABBILDUNG 2:**
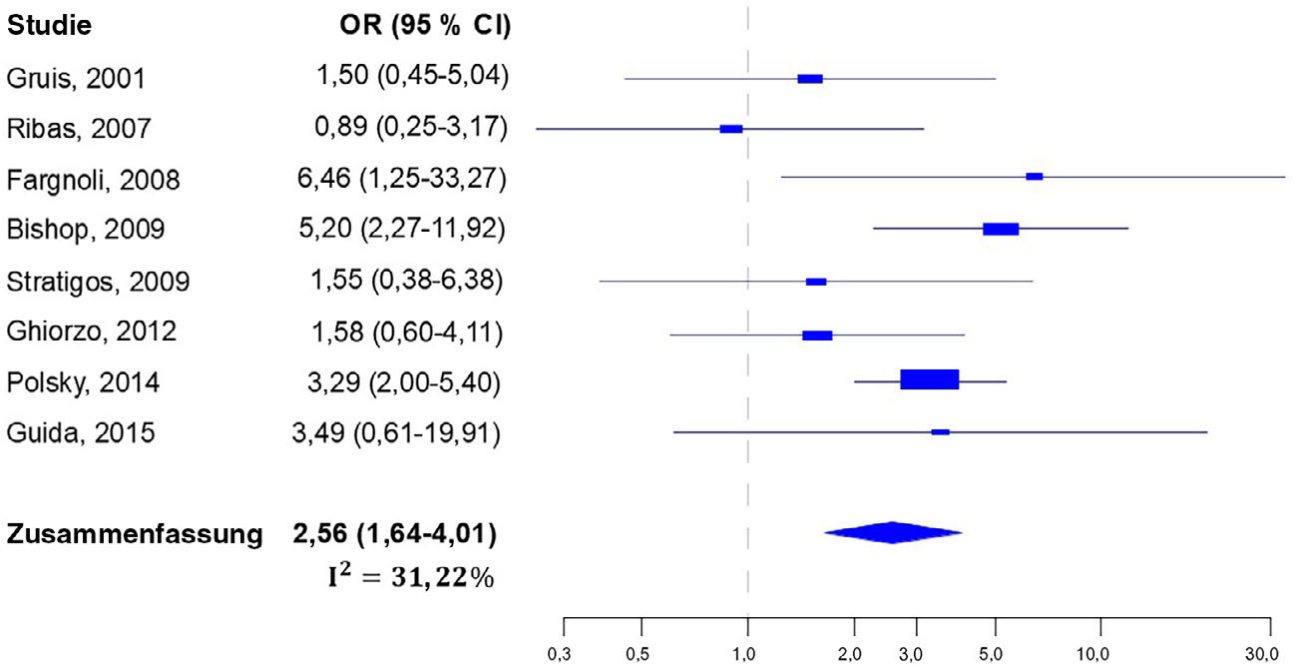
Walddiagramm der angepassten ORs für den Zusammenhang zwischen gewöhnlichen Nävi und dem Melanomrisiko in der jüngeren Altersgruppe. Personen mit einer Anzahl gewöhnlicher Nävi unterhalb bzw. oberhalb des studienspezifischen Medians wurden in der Kontrollgruppe verglichen.

Die zusammengefasste OR (sOR) für die Assoziation zwischen dem Vorliegen vieler gewöhnlicher Nävi und Melanomen bei jüngeren Personen (< 40 Jahre) war positiv und signifikant: sOR = 2,56 (95 % CI 1,64–4,01), bei einer geringen Heterogenität von 31,22 % zwischen den Studien (Abbildung 2).

**ABBILDUNG 3:**
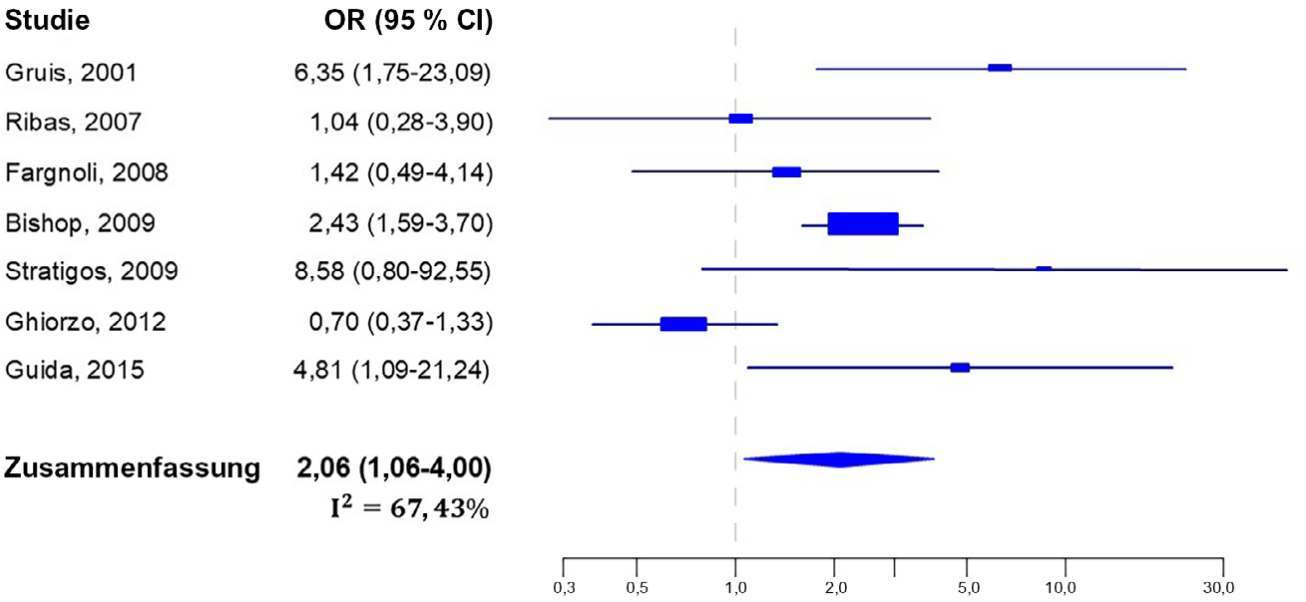
Walddiagramm der angepassten ORs für den Zusammenhang zwischen gewöhnlichen Nävi und dem Melanomrisiko in der älteren Altersgruppe. Personen mit einer Anzahl gewöhnlicher Nävi unterhalb bzw. oberhalb des studienspezifischen Medians wurden in der Kontrollgruppe verglichen.

Für die ältere Subgruppe zeigte sich, wie in Abbildung 3 dargestellt, dass die sOR = 2,06 (95 % CI 1,06–4,00) auf eine signifikante Verdopplung des Melanomrisikos bei Personen mit einer hohen Anzahl gewöhnlicher Nävi hinwies; allerdings wurde eine starke Heterogenität festgestellt (I^2^ = 67,43 %). Ohne die Daten von Ghiorzo^32^, ^33^, ^34^, ^35^ betrug der Wert von I^2^ = 0 und die zusammengefasste Schätzung ergab eine sOR von 2,48 (95 % CI 1,75–3,50) (Online‐Ergänzungsabbildung S1).

Eine Metaregressionsanalyse der Gesamtstichprobe, mit der die Unterschiede zwischen den beiden Subgruppen (junge vs. ältere Menschen) untersucht werden sollten, ergab keinen signifikanten Unterschied zwischen den Altersgruppen hinsichtlich des Melanomrisikos im Zusammenhang mit gewöhnlichen Nävi (p = 0,57).

Anschließend führten wir eine Sensitivitätsanalyse durch, wobei wir die Studien von Ribas und Stratigos^23^, ^29^, ^30^, ^31^ ausschlossen, da sie eine unerwartet gleiche mediane Anzahl gewöhnlicher Nävi für jüngere und ältere Menschen in der Kontrollgruppe aufwiesen.

Nach diesen Ausschlüssen stieg die sOR für die Assoziation zwischen dem Vorliegen vieler gewöhnlicher Nävi und Melanomen bei jungen Menschen auf 3,11 (95 % CI 2,13–4,54) (Online‐Ergänzungsabbildung S2), wobei die Heterogenität abnahm (I^2^ = 6,13 %). Für die ältere Gruppe war jedoch die sOR mit 2,09 (95 % CI 0,96–4,53) nicht mehr statistisch signifikant (Online‐Ergänzungsabbildung S3). Die Heterogenität betrug 75,7 % und nach Eliminierung der Studie von Ghiorzo^32^, ^33^, ^34^, ^35^ betrug sie 0 mit einem sOR von 2,57 (95 % CI 1,78–3,69). Dennoch zeigte die Metaregression der Gesamtstichprobe keine signifikanten Veränderungen des Melanomrisikos in Verbindung mit einer hohen Anzahl gewöhnlicher Nävi nach Altersgruppen (p = 0,34).

Anschließend führten wir eine zweite Sensitivitätsanalyse durch, in die wir die Kohorten von Menin und Puig^38^, ^39^, ^40^, ^41^, ^42^, ^43^ einbezogen, die zuvor aufgrund des Nichtzutreffens der Einschlusskriterien aus der Studie ausgeschlossen worden waren (Online‐Material). Die sOR für die Assoziation zwischen dem Vorliegen vieler gewöhnlicher Nävi und Melanomen bei jüngeren Menschen (< 40 Jahre) betrug 1,68 (95 % CI 0,89–3,16) (Ergänzungsabbildung S4). Der Zusammenhang zwischen einer hohen Anzahl gewöhnlicher Nävi und dem Melanomrisiko war statistisch nicht signifikant (p = 0,11); die Heterogenität zwischen den Studien betrug 69,59 %. Bei älteren Menschen deutete die zusammengefasste Schätzung auf ein doppelt so hohes Risiko hin: sOR = 2,00 (95 % CI 1,09–3,68; I^2^ = 61,38 %) (Online‐Ergänzungsabbildung S5).

Da die Kontrollgruppen in den verschiedenen Studien variierten, führten wir schließlich eine Subgruppenanalyse durch, bei der wir die Studien in solche mit hospitalisierten Kontrollen und solche mit Kontrollen aus der Allgemeinbevölkerung oder anderweitig gesunden Personen unterteilten. Wir stellten fest, dass bei Jüngeren kein signifikanter Unterschied zwischen den beiden Kontrollgruppen bestand (p = 0,38). Bei älteren Personen zeigten Studien mit hospitalisierten Kontrollpersonen jedoch eine signifikante sOR von 2,65 (95 % CI 1,83–3,85), während solche mit Kontrollpersonen aus der Allgemeinbevölkerung eine nicht signifikante sOR von 0,76 (95 % CI 0,42–1,35) ergaben. In diesem Fall war der Einfluss des Studiendesigns auf die Assoziation zwischen der Nävuszahl und dem Risiko, an einem Melanom zu erkranken, statistisch signifikant (p < 0,001).

### Atypische Nävi

Für atypische Nävi wurden sieben Studien ^20–22,24–35,37,44,45^ mit 2.459 Fällen und 1.740 Kontrollen ausgewählt: 602 bzw. 687 Fälle und 297 bzw. 674 Kontrollen bei jüngeren (< 40 Jahre) bzw. älteren Menschen (über 60 Jahre) (Tabelle 1).

**ABBILDUNG 4:**
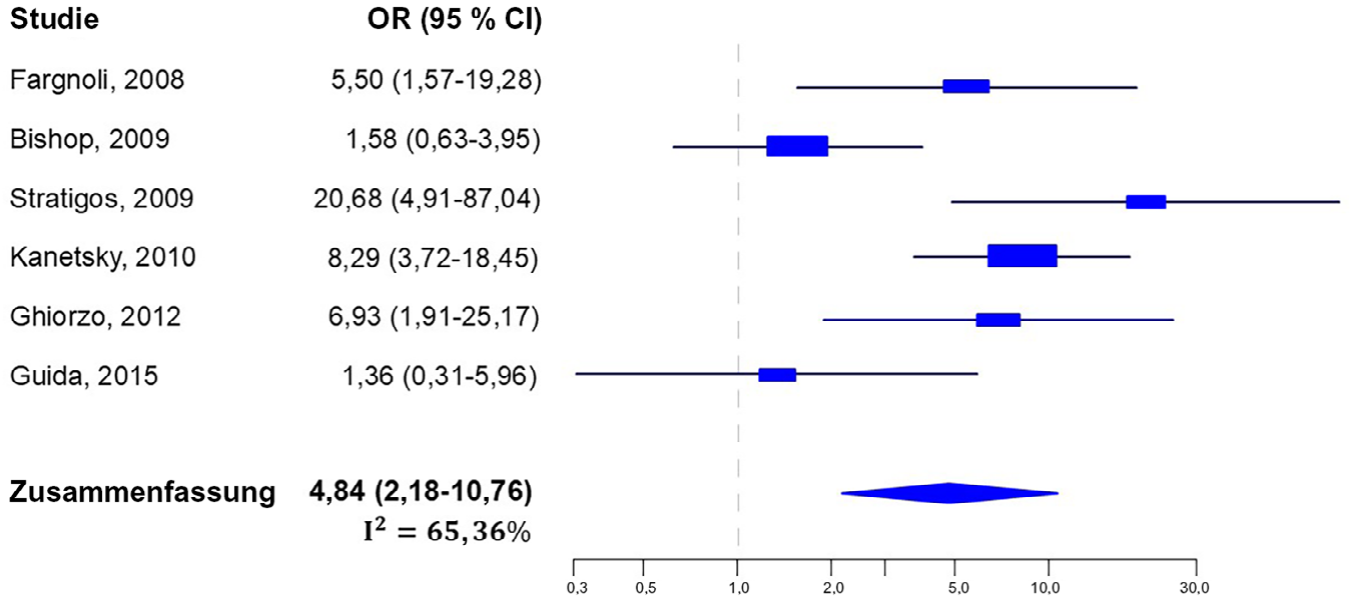
Walddiagramm der angepassten ORs für den Zusammenhang zwischen atypischen Nävi und dem Melanomrisiko in der jüngeren Altersgruppe. Es wurden Vorliegen und Nichtvorlegen atypischer Nävi verglichen.

Bei jüngeren Personen war die sOR = 4,84 (95 % CI 2,18–10,76) für das Vorliegen von mindestens einem atypischen Nävus signifikant, jedoch bestand eine hohe Heterogenität zwischen den Studien (I^2^ = 65,36 %) (Abbildung 4). Eine von Bishop^28^ durchgeführte Studie war hauptsächlich für die Heterogenität verantwortlich. Nach Ausschluss dieser Studie blieb die Assoziation zwischen dem Vorliegen eines atypischen Nävus und dem Melanomrisiko signifikant: OR = 6,48 (95 % CI 3,21–13,09) mit einem I^2^ von 40,56 % (Online‐Ergänzungsabbildung S6).

Die Online‐Ergänzungstabellen S4a (jüngere Gruppe) und S4b (ältere Gruppe) listen die rohen und bereinigten ORs für jede Studie auf, wobei eine Reihe signifikanter Störvariablen berücksichtigt wurde. Die Einbeziehung der Untersuchungen von Menin und Puig ^38,39–43^ in die Analyse ergab ebenfalls eine ähnliche Gesamtschätzung von sOR = 5,71 (95 % CI 2,21–14,72), jedoch stieg die Heterogenität signifikant an (I^2^ = 81,0 %) (Online‐Ergänzungsabbildung S7).

**ABBILDUNG 5:**
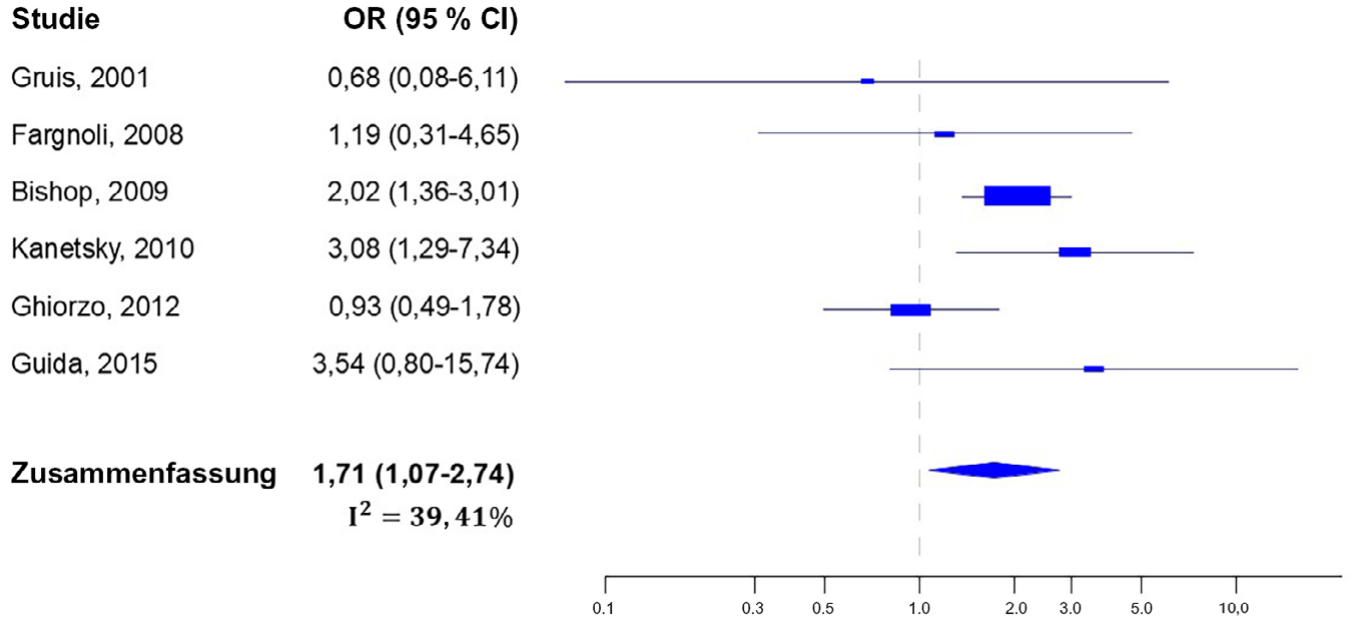
Walddiagramm der angepassten ORs für den Zusammenhang zwischen atypischen Nävi und dem Melanomrisiko in der älteren Altersgruppe. Es wurden Vorliegen und Nichtvorlegen atypischer Nävi verglichen.

Bei älteren Personen war das Risiko, an einem Melanom zu erkranken, das mit einem oder mehreren atypischen Nävi assoziiert war, viel geringer als bei der jüngeren Altersgruppe (OR = 1,71; 95 % CI 1,07–2,74) mit einer geringen Heterogenität (I^2^ = 39,41 %) (Abbildung 5). Ohne die Studie von Ghiorzo ^32–35^ betrug die Heterogenität 0 mit einem Wert der geschätzten OR von 2,09 (95 % CI 1,49–2,93).

In der Gesamtanalyse war die Variation des Melanomrisikos in Bezug auf atypische Nävi zwischen der jüngeren und der älteren Gruppe statistisch signifikant (p = 0,02).

## DISKUSSION

Das Melanomrisiko wird maßgeblich durch die Eigenschaften der Nävi beeinflusst, wobei sowohl ihre Anzahl als auch ihre Art als unabhängige Risikofaktoren wirken. Es besteht eine starke Assoziation zwischen der Gesamtzahl der Nävi am Körper und dem Melanomrisiko.^5^ Mehr als elf Nävi an einem Arm sind ein Prädiktor für eine hohe TBNC.^46^ Während die Wahrscheinlichkeit, dass sich ein einzelner Nävus sich zu einem Melanom entwickelt, gering ist, sind mehrere gewöhnliche und atypische Nävi ein Indikator für eine höhere Anfälligkeit für Melanome.^47^

Das Verständnis des Melanomrisikos in verschiedenen Altersgruppen ist ausschlaggebend, da Melanome im Vergleich zu vielen anderen Krebsarten disproportional häufig jüngere Menschen betreffen und die Identifizierung von Hochrisikogruppen effektivere und gezielte Sekundärpräventionsmaßnahmen unterstützen kann.

Die Entwicklung von Nävi im individuellen Lebensverlauf liefert Erkenntnisse über die Seneszenz von Melanozyten.^48^ Interessanterweise haben Personen mit hoher Nävuszahl tendenziell eine günstigere Melanom‐Prognose, einschließlich besserer Überlebensrate, was möglicherweise auf eine reduzierte zelluläre Seneszenz sowohl in Melanozyten als auch in Immunzellen zurückzuführen ist.^49^

Nävi sind wachstumsgehemmte, klonale Neoplasien von Melanozyten, die durch Mutationen im Mitogen‐aktivierten Proteinkinase (MAPK)‐Signalweg verursacht werden, am häufigsten durch die aktivierende BRAF‐V600E‐Mutation.^50^ Die Nävuszahl erreicht in der vierten Lebensdekade ihren Höhepunkt, was auf eine Kombination aus verminderter Neubildung von Nävi und Regression bestehender Nävi zurückzuführen ist.^48^ Dermatoskopische Untersuchungen zeigen, dass Nävi einem typischen Lebenszyklus folgen: In der Kindheit erscheinen sie globulär, gehen im Erwachsenenalter in retikuläre Muster über und werden schließlich zu zusammengesetzten oder dermalen Nävi, bevor sie bei älteren Erwachsenen verschwinden, insbesondere in Regionen außerhalb des Kopf‐ und Halsbereichs.^51^, ^52^, ^53^, ^54^

Das Verständnis zur klinischen Regression von Nävi ist nicht gut, es ist jedoch bekannt, dass Immunsignale und genetische Faktoren wie BRAF, CDKN2A und telomerbezogene Gene beteiligt sind.^55^ Die Telomerlänge steht in Zusammenhang mit der Nävuszahl, wobei Personen mit homozygoten Funktionsverlust‐Mutationen in P16 (CDKN2A‐null) manchmal zahlreiche atypische Nävi entwickeln, die mit zunehmendem Alter bestehen bleiben und die Anfälligkeit für Melanome und andere Krebsarten erhöhen.^56^

Eine systematische Überprüfung^50^ von 708 Studien ergab nur zwei bevölkerungsbasierte Längsschnittstudien, die die Einschlusskriterien für die Zählung von Nävi bei Erwachsenen mit einer Baseline und mindestens einer Nachuntersuchung erfüllten.^57^, ^58^ Ribero et al. führten eine 15‐jährige Längsschnittstudie durch, die zeigte, dass die TBNC laut Querschnittsdaten mit zunehmendem Alter abnimmt.^16^ Längsschnittdaten deuteten hingegen auf einen leichten Anstieg der TBNC bei älteren Personen hin, möglicherweise aufgrund einer Fehlklassifizierung von Lentigines solares und seborrhoischen Keratosen als Nävi. Der in Querschnittsstudien beobachtete Rückgang der TBNC lässt sich teilweise durch einen Kohorteneffekt erklären, wie von Plasmeijer et al.^50^ vorgeschlagen.

Auf der Basis dieser früheren Erkenntnisse war das Ziel unserer Studie, den Zusammenhang zwischen der Nävuszahl und dem Melanomrisiko in verschiedenen Altersgruppen weiter zu untersuchen. Unsere Ergebnisse zeigten, dass beim Vergleich jüngerer und älterer Personen eine hohe Anzahl gewöhnlicher Nävi im Vergleich zu einer geringen Anzahl nicht signifikant mit einem erhöhten Melanomrisiko assoziiert war (p = 0,57). Das mit einer hohen Anzahl gewöhnlicher Nävi verbundene Melanomrisiko erwies sich jedoch in beiden Gruppen als signifikant, mit einer OR von 2,56 bei jüngeren und 2,06 bei älteren Personen. Die Anpassung für atypische Nävi in der Analyse der gewöhnlichen Nävi änderte nichts an den Ergebnissen (angepasster p‐Wert = 0,39); dies gilt in ähnlicher Weise, wenn auch in geringerem Maße, für die Analyse atypischer Nävi. Obwohl die Nävuszahl klinisch gesehen mit zunehmendem Alter abzunehmen scheint, konnten Längschnittstudien und Metaanalysen den genauen Zeitpunkt im Leben, zu dem dies geschieht, nicht genau bestimmen, was wahrscheinlich den Mangel an signifikanten Unterschieden im Melanomrisiko zwischen den Altersgruppen erklärt.

Bei atypischen Nävi war das Melanomrisiko bei jüngeren Personen höher als bei älteren (sOR = 4,84 vs. 1,71). Dies deutet darauf hin, dass das Risiko, ein Melanom zu entwickeln, bei Vorliegen eines atypischen Nävus im Vergleich zu keinem atypischen Nävus bei Personen unter 40 Jahren viel höher ist als bei Personen über 60 Jahren (p‐Wert = 0,02). Dies impliziert nicht, dass sich ein einzelner atypischer Nävus mit hoher Wahrscheinlichkeit zu einem Melanom entwickelt; jedoch ist das Vorliegen atypischer Nävi ein stärkerer Prädiktor für das Melanomrisiko bei jüngeren Menschen.

Der prädiktive Wert atypischer Nävi nimmt bei älteren Menschen ab, möglicherweise aufgrund von Unterschieden in den Melanom‐Entstehungswegen. Nach dem Zwei‐Pfad‐Modell von Whiteman^59^ kann ein Melanom auf unterschiedlichen Wegen entstehen: Personen mit zahlreichen Nävi entwickeln eher früher im Leben und bei geringerer kumulativer Sonnenexposition nävusassoziierte Melanome; bei älteren Menschen hingegen entwickeln sich eher nach kontinuierlicher Sonnenexposition *De‐novo*‐Melanome, wodurch die relative Bedeutung der Nävuszahl als Risikofaktor in dieser Population abnimmt.

Auch präventive Untersuchungen könnten diese Trends beeinflusst haben. Historisch wurden atypische Nävi bei älteren Patienten häufig ohne dermatoskopische Abklärung zur Verhinderung einer malignen Transformation entfernt, wodurch sich das wahrgenommene Risiko möglicherweise veränderte, da sie entfernt wurden, bevor sie in klinische Studien einbezogen werden konnten. Diese Hypothese konnte jedoch aufgrund fehlender retrospektiver Daten zur Narbenanzahl in Fällen und Kontrollen nicht bestätigt werden.

### Stärken und Grenzen

Diese Studie bestand aus einer gepoolten Analyse.^18^ Es wurden Daten aus über 30 Untersuchungen im M‐SKIP‐Datensatz gesammelt.^13^ Dies machte die Durchführung robuster Subgruppenanalysen nach Alter und Nävustyp möglich und erleichterte die Untersuchung seltener Expositionen sowie die Formulierung neuer Hypothesen. Durch die Integration von Daten aus mehreren Studien wurde die statistische Aussagekraft erhöht, was eine präzisere Abschätzung des Melanomrisikos ermöglichte. Altersbedingte Unterschiede konnten hervorgehoben und quantifiziert und gleichzeitig die Gesamtheterogenität minimiert werden. Darüber hinaus ermöglichte die Standardisierung der Variablen über verschiedene Studien hinweg Anpassungen auf der Grundlage mehrerer Störfaktoren wie Geschlecht, Sonnenexposition und MC1R‐Mutationen, wodurch die Zuverlässigkeit der Ergebnisse weiter verbessert wurde.

Das retrospektive Design der Studie stellte jedoch eine Herausforderung dar, da die Ausgangsstudien unterschiedliche Forschungsziele hatten. Ihre Methoden zur Datenerhebung variierten, sodass eine Standardisierung erforderlich war, um einen kohärenten Datensatz zu erstellen, was zu OR‐Anpassungen für verschiedene Gruppen von Störfaktoren zwischen den Studien führte. Darüber hinaus verhinderten Unterschiede in der Verteilung der Nävi zwischen den Studien die Definition eines einheitlichen Medians, sodass die mediane Nävuszahl in jeder Kontrollgruppe als Referenz verwendet wurde. Diese Variabilität könnte die Ergebnisse beeinflusst haben, obwohl Anstrengungen unternommen wurden, die Datenanalyse zu standardisieren.

## SCHLUSSFOLGERUNGEN

Insgesamt liefert die Studie wertvolle Erkenntnisse über altersbezogene Schwankungen des Melanomrisikos auf der Grundlage der Nävuszahl und atypischer Nävi. Diese Ergebnisse unterstreichen die Bedeutung einer altersspezifischen Risikobewertung und zeigen die Notwendigkeit auf, die genetische Prädisposition und ein verbessertes langzeitliches Monitoring der Nävus‐Entwicklung zu integrieren, um gezieltere Strategien der Sekundärprävention zu unterstützen.

## DANKSAGUNG

Wir danken den an der M‐SKIP‐Studiengruppe teilnehmenden Zentren für die Bereitstellung der Originaldatensätze. Die M‐SKIP‐Studiengruppe besteht aus folgenden Mitgliedern: PI: Sara Raimondi (European Institute of Oncology, Mailand, Italien); Beiratsmitglieder: Philippe Autier (International Prevention Research Institute, Lyon, Frankreich), Maria Concetta Fargnoli (University of L'Aquila, Italien), José C. García‐Borrón (University of Murcia, Spanien), Jiali Han (Brigham and Women's Hospital und Harvard Medical School, Boston, MA, USA), Peter A. Kanetsky (Perelman School of Medicine at the University of Pennsylvania, Philadelphia, PA, USA), Maria Teresa Landi (National Cancer Institute, NIH, Bethesda, MD, USA), Julian Little (University of Ottawa, Kanada), Julia Newton‐Bishop (University of Leeds, UK), Francesco Sera (UCL Institute of Child Health, London, UK); Berater: Saverio Caini (ISPO, Florenz, Italien), Sara Gandini und Patrick Maisonneuve (European Institute of Oncology, Mailand, Italien); Teilnehmende Forscher: Albert Hofman, Manfred Kayser, Fan Liu, Tamar Nijsten und Andre G. Uitterlinden (Erasmus MC University Medical Center, Rotterdam, Niederlande), Rajiv Kumar und Dominique Scherer (German Cancer Research Center, Heidelberg, Deutschland), Eduardo Nagore (Instituto Valenciano de Oncologia, Valencia, Spanien), Johan Hansson und Veronica Hoiom (Karolinska Institutet, Stockholm, Schweden), Paola Ghiorzo und Lorenza Pastorino (University of Genoa, Italien), Nelleke A. Gruis (Leiden University Medical Center, Niederlande), Terry Dwyer (Murdoch Children's Research Institute, Victoria, Australien), Leight Blizzard und Jennifer Cochrane (Menzies Research Institute, Hobart, Australien), Ricardo Fernandez‐de‐Misa (Hospital Universitario Nuestra Señora de Candelaria, Santa Cruz de Tenerife, Spanien), Wojciech Branicki (Institute of Forensic Research, Krakow, Polen), Tadeusz Debniak (Pomeranian Medical University, Polabska, Polen), Niels Morling und Peter Johansen (University of Copenhagen, Dänemark), Ruth Pfeiffer (National Cancer Institute, NIH, Bethesda, MD, USA), Giuseppe Palmieri (Istituto di Chimica Biomolecolare, CNR, Sassari, Italien), Gloria Ribas (Fundacion Investigation Hospital Clinico Universitario de Valencia‐ INCLIVA, Spanien), Alexander Stratigos und Katerina Kypreou (University of Athens, Andreas Sygros Hospital, Athens, Griechenland), Anne Bowcock und Lynn Cornelius (Washington University, St. Louis, MO, USA), M. Laurin Council (St. Louis University, St. Louis, MO, USA), Tomonori Motokawa (POLA Chemical Industries, Yokohama, Japan), Sumiko Anno (Shibaura Institute of Technology, Tokyo, Japan), Per Helsing und Per Arne Andresen (Oslo University Hospital, Norwegen), Terence H. Wong (University of Edinburgh, UK), und die Genes, Environment and Melanoma (GEM‐) Studiengruppe.

Die Teilnehmer an der GEM‐Studiengruppe sind wie folgt: Koordinierendes Zentrum, Memorial Sloan‐Kettering Cancer Center, New York, NY, USA: Marianne Berwick (PI, aktuell University of New Mexico), Colin Begg (Co‐PI), Irene Orlow (Co‐Investigator), Urvi Mujumdar (Projektkoordinator), Amanda Hummer (Biostatistikerin), Klaus Busam (Dermatopathologe), Pampa Roy (Labortechnikerin), Rebecca Canchola (Labortechnikerin), Brian Clas (Labortechniker), Javiar Cotignola (Labortechnikerin), Yvette Monroe (Interviewerin). Studienzentren: The University of Sydney und The Cancer Council New South Wales, Sydney (Australien): Bruce Armstrong (PI), Anne Kricker (Co‐PI), Melisa Litchfield (Studienkoordinatorin). Menzies Centre for Population Health Research, University of Tasmania, Hobart (Australien): Terence Dwyer (PI), Paul Tucker (Dermatopathologe), Nicola Stephens (Studienkoordinatorin). British Columbia Cancer Agency, Vancouver (Kanada): Richard Gallagher (PI), Teresa Switzer (Koordinator). Cancer Care Ontario, Toronto (Kanada): Loraine Marrett (PI), Beth Theis (Co‐Investigator), Lynn From (Dermatopathologin), Noori Chowdhury (Koordinator), Louise Vanasse (Koordinatorin), Mark Purdue (Forschungsbeauftragter). David Northrup (CATI‐Manager). Centro per la Prevenzione Oncologia Torino, Piemonte (Italien): Roberto Zanetti (PI), Stefano Rosso (Datenmanager), Carlotta Sacerdote (Koordinatorin). University of California, Irvine (USA): Hoda Anton‐Culver (PI), Nancy Leighton (Koordinatorin), Maureen Gildea (Datenmanagerin). University of Michigan, Ann Arbor (USA): Stephen Gruber (PI), Joe Bonner (Datenmanager), Joanne Jeter (Koordinatorin). New Jersey Department of Health and Senior Services, Trenton (USA): Judith Klotz (PI), Homer Wilcox (Co‐PI), Helen Weiss (Koordinatorin). University of North Carolina, Chapel Hill (USA): Robert Millikan (PI), Nancy Thomas (Co‐Investigator), Dianne Mattingly (Koordinatorin), Jon Player (Labortechniker), Chiu‐Kit Tse (Datenanalytiker). University of Pennsylvania, Philadelphia, PA (USA): Timothy Rebbeck (PI), Peter Kanetsky (Co‐Investigator), Amy Walker (Labortechnikerin), Saarene Panossian (Laboratortechniker). Berater: Harvey Mohrenweiser, University of California, Irvine, Irvine, CA (USA); Richard Setlow, Brookhaven National Laboratory, Upton, NY (USA).

Das European Institute of Oncology (IEO) wird teilweise vom italienischen Gesundheitsministerium (mit Mitteln aus den “Ricerca Corrente”‐ und “5 × 1000”‐Fonds) unterstützt.

## INTERESSENKONFLIKT

Keiner.

## Supporting information



Supplementary information
