# Supplementary material for: Altersspezifisches Melanomrisiko im Zusammenhang mit melanozytären Nävi: Eine gepoolte Analyse aus dem M‐SKIP‐Projekt
Source: J Dtsch Dermatol Ges. 2026 Sep 8;24(9):1221–31. [Article in German] doi: 10.1111/ddg.15992_g (PMC13551281; doi:10.1111/ddg.15992_g)
Supplement: Supplementary file 1 [file DDG-24-1221-s001.docx]

**SUPPLEMENTARY**

EXPLANATIONS FOR STUDY EXCLUSION

This section explains the reasons behind the exclusion of certain studies from the analysis.

Some studies initially included in the research were later excluded from the main analysis because they did not fully meet the inclusion criteria established at the outset. Conversely, other studies that met the inclusion criteria were included in the main analysis but were subsequently excluded through sensitivity analyses to determine whether their exclusion would impact the results.

**Studies by Menin and Puig**

The studies by Menin and Puig were excluded from the main analysis because the populations studied were at a higher risk of melanoma. However, a sensitivity analysis was performed including these studies to assess whether their inclusion would affect the results.

Specifically, Puig’s study is a family research study where the controls, who are healthy family members of melanoma patients, are individuals with an elevated risk of developing melanoma. Similarly, in Menin’s study all the cases analysed had an increased likelihood of developing melanoma due to having at least one family member with the condition.

**Studies by Stratigos and Ribas**

Both studies reported the same median number of common nevi among younger and older subjects. This may be attributed to the fact that the studies were conducted in Greece and Spain, respectively, where it was observed that the general population has a low number of nevi. These studies were included in the main analysis but were subsequently excluded in a sensitivity analysis.

**Ghiorzo’s study**

In Ghiorzo’s study, all cases were examined by a doctor, while only half of the controls underwent a medical examination; the remaining controls performed a self-examination. Furthermore, since this study was conducted by a geneticist, it is possible that the controls were selected from individuals with a higher genetic risk of melanoma. This study was included in the main analysis; however, for the subgroup of older participants, it was later excluded in a sensitivity analysis to evaluate its contribution to the high heterogeneity observed in the model.

**Table S1.** Descriptive statistics on Melanoma and Common naevi

|  | **MELANOMA** | | | | **COMMON NAEVI** | | | |
| --- | --- | --- | --- | --- | --- | --- | --- | --- |
| **Characteristic** | **Overall,  N = 5.017** | **Melanoma cases,  N = 2.674** | **Controls,  N = 2.343** | **p-value*** | **Overall,  N = 5.017** | **>median,  N = 2.005** | **≤median,  N = 3.012** | **p-value*** |
| Common Nevi |  |  |  | <0.001 |  |  |  |  |
| ≤median | 3.012 (60%) | 1.332 (50%) | 1.680 (72%) |  |  |  |  |  |
| >median | 2.005 (40%) | 1.342 (50%) | 663 (28%) |  |  |  |  |  |
| Atypical Nevi |  |  |  | <0.001 |  |  |  | <0.001 |
| no | 1.923 (62%) | 811 (48%) | 1.112 (79%) |  | 1.923 (62%) | 705 (44%) | 1.218 (82%) |  |
| yes | 1.176 (38%) | 874 (52%) | 302 (21%) |  | 1.176 (38%) | 901 (56%) | 275 (18%) |  |
| Missing | 1.918 | 989 | 929 |  | 1.918 | 399 | 1.519 |  |
| Age |  |  |  | <0.001 |  |  |  | <0.001 |
| <40 | 1.038 (21%) | 605 (23%) | 433 (18%) |  | 1.038 (21%) | 491 (24%) | 547 (18%) |  |
| 40-60 | 2.831 (56%) | 1.536 (57%) | 1.295 (55%) |  | 2.831 (56%) | 1.008 (50%) | 1.823 (61%) |  |
| >60 | 1.148 (23%) | 533 (20%) | 615 (26%) |  | 1.148 (23%) | 506 (25%) | 642 (21%) |  |
| Intermittent sun exposure |  |  |  | 0.037 |  |  |  | 0.039 |
| low | 2.477 (53%) | 1.366 (54%) | 1.111 (51%) |  | 2.477 (53%) | 968 (51%) | 1.509 (54%) |  |
| high | 2.207 (47%) | 1.150 (46%) | 1.057 (49%) |  | 2.207 (47%) | 928 (49%) | 1.279 (46%) |  |
| Missing | 333 | 158 | 175 |  | 333 | 109 | 224 |  |
| Continuous sun exposure |  |  |  | <0.001 |  |  |  | <0.001 |
| ≤0 | 2.306 (49%) | 1.029 (42%) | 1.277 (57%) |  | 2.306 (49%) | 680 (36%) | 1.626 (58%) |  |
| >0 | 2.362 (51%) | 1.400 (58%) | 962 (43%) |  | 2.362 (51%) | 1.190 (64%) | 1.172 (42%) |  |
| Missing | 349 | 245 | 104 |  | 349 | 135 | 214 |  |
| Hair colour |  |  |  | <0.001 |  |  |  | 0.6 |
| red | 349 (7.0%) | 262 (9.9%) | 87 (3.7%) |  | 349 (7.0%) | 146 (7.3%) | 203 (6.8%) |  |
| light | 2.345 (47%) | 1.271 (48%) | 1.074 (46%) |  | 2.345 (47%) | 944 (47%) | 1.401 (47%) |  |
| dark | 2.294 (46%) | 1.118 (42%) | 1.176 (50%) |  | 2.294 (46%) | 900 (45%) | 1.394 (46%) |  |
| Missing | 29 | 23 | 6 |  | 29 | 15 | 14 |  |

| Eye colour |  |  |  | <0.001 |  |  |  | 0.003 |
| --- | --- | --- | --- | --- | --- | --- | --- | --- |
| light | 2.896 (64%) | 1.569 (66%) | 1.327 (61%) |  | 2.896 (64%) | 1.105 (67%) | 1.791 (62%) |  |
| dark | 1.631 (36%) | 792 (34%) | 839 (39%) |  | 1.631 (36%) | 549 (33%) | 1.082 (38%) |  |
| Missing | 490 | 313 | 177 |  | 490 | 351 | 139 |  |
| Phototype (Fitzpatrick classification) |  |  |  | <0.001 |  |  |  | 0.6 |
| low | 1.959 (41%) | 1.173 (45%) | 786 (36%) |  | 1.959 (41%) | 784 (40%) | 1.175 (41%) |  |
| medium | 2.832 (59%) | 1.441 (55%) | 1.391 (64%) |  | 2.832 (59%) | 1.154 (60%) | 1.678 (59%) |  |
| high | 0 (0%) | 0 (0%) | 0 (0%) |  | 0 (0%) | 0 (0%) | 0 (0%) |  |
| Missing | 226 | 60 | 166 |  | 226 | 67 | 159 |  |
| Mutation of MC1R gene |  |  |  | <0.001 |  |  |  | <0.001 |
| MC1R=0 | 1.305 (26%) | 542 (21%) | 763 (33%) |  | 1.305 (26%) | 454 (23%) | 851 (28%) |  |
| MC1R≠0 | 3.650 (74%) | 2.100 (79%) | 1.550 (67%) |  | 3.650 (74%) | 1.513 (77%) | 2.137 (72%) |  |
| Missing | 62 | 32 | 30 |  | 62 | 38 | 24 |  |
| Gender |  |  |  | 0.2 |  |  |  | 0.059 |
| male | 2.131 (42%) | 1.114 (42%) | 1.017 (43%) |  | 2.131 (42%) | 884 (44%) | 1.247 (41%) |  |
| female | 2.886 (58%) | 1.560 (58%) | 1.326 (57%) |  | 2.886 (58%) | 1.121 (56%) | 1.765 (59%) |  |
| Family history of melanoma |  |  |  | >0.9 |  |  |  | 0.8 |
| none | 4.149 (89%) | 2.281 (89%) | 1.868 (89%) |  | 4.149 (89%) | 1.684 (89%) | 2.465 (89%) |  |
| yes | 515 (11%) | 282 (11%) | 233 (11%) |  | 515 (11%) | 212 (11%) | 303 (11%) |  |
| Missing | 353 | 111 | 242 |  | 353 | 109 | 244 |  |

*Continuous variables are described by using the median and the interquartile range (IQR), while the categorical variables are summarized through the absolute frequencies and the column percentages*

** Fisher's test and Pearson's Chi-squared test for categorical variables, and Wilcoxon rank sum test for quantitative variables*

**Table S2.** Descriptive statistics on Melanoma and Atypical naevi

|  | **MELANOMA** | | | | **ATYPICAL NAEVI** | | | |
| --- | --- | --- | --- | --- | --- | --- | --- | --- |
| **Characteristic** | **Overall, N = 4.199** | **Melanoma cases,  N = 2.459** | **Controls,  N =1.740** | **p-value*** | **Overall,  N = 4.199** | **Yes,  N = 1.581** | **No,  N = 2.618** | **p-value*** |
| Atypical Nevi |  |  |  | <0.001 |  |  |  |  |
| no | 2.618 (62%) | 1.223 (50%) | 1.395 (80%) |  |  |  |  |  |
| yes | 1.581 (38%) | 1.236 (50%) | 345 (20%) |  |  |  |  |  |
| Common Nevi |  |  |  | <0.001 |  |  |  | <0.001 |
| ≤median | 1.688 (52%) | 724 (41%) | 964 (64%) |  | 1.688 (52%) | 321 (27%) | 1.367 (67%) |  |
| >median | 1.553 (48%) | 1.022 (59%) | 531 (36%) |  | 1.553 (48%) | 875 (73%) | 678 (33%) |  |
| Unknown | 958 | 713 | 245 |  | 958 | 385 | 573 |  |
| Age |  |  |  | <0.001 |  |  |  | <0.001 |
| <40 | 899 (21%) | 602 (24%) | 297 (17%) |  | 899 (21%) | 416 (26%) | 483 (18%) |  |
| 40-60 | 1.939 (46%) | 1.170 (48%) | 769 (44%) |  | 1.939 (46%) | 772 (49%) | 1.167 (45%) |  |
| >60 | 1.361 (32%) | 687 (28%) | 674 (39%) |  | 1.361 (32%) | 393 (25%) | 968 (37%) |  |
| Intermittent sun exposure |  |  |  | 0.3 |  |  |  | 0.063 |
| low | 2.012 (49%) | 1.190 (50%) | 822 (48%) |  | 2.012 (49%) | 786 (51%) | 1.226 (48%) |  |
| high | 2.102 (51%) | 1.212 (50%) | 890 (52%) |  | 2.102 (51%) | 762 (49%) | 1.340 (52%) |  |
| Unknown | 85 | 57 | 28 |  | 85 | 33 | 52 |  |
| Continuous sun exposure |  |  |  | <0.001 |  |  |  | <0.001 |
| ≤0 | 1.883 (48%) | 1.014 (44%) | 869 (53%) |  | 1.883 (48%) | 513 (34%) | 1.370 (56%) |  |
| >0 | 2.063 (52%) | 1.304 (56%) | 759 (47%) |  | 2.063 (52%) | 981 (66%) | 1.082 (44%) |  |
| Unknown | 253 | 141 | 112 |  | 253 | 87 | 166 |  |
| Hair colour |  |  |  | <0.001 |  |  |  | <0.001 |
| red | 323 (7.8%) | 249 (10%) | 74 (4.3%) |  | 323 (7.8%) | 153 (9.8%) | 170 (6.5%) |  |
| light | 1.632 (39%) | 975 (40%) | 657 (38%) |  | 1.632 (39%) | 658 (42%) | 974 (37%) |  |
| dark | 2.210 (53%) | 1.210 (50%) | 1.000 (58%) |  | 2.210 (53%) | 755 (48%) | 1.455 (56%) |  |
| Unknown | 34 | 25 | 9 |  | 34 | 15 | 19 |  |

| Eye colour |  |  |  | <0.001 |  |  |  | <0.001 |
| --- | --- | --- | --- | --- | --- | --- | --- | --- |
| light | 2.271 (61%) | 1.372 (64%) | 899 (58%) |  | 2.271 (61%) | 875 (68%) | 1.396 (58%) |  |
| dark | 1.432 (39%) | 772 (36%) | 660 (42%) |  | 1.432 (39%) | 420 (32%) | 1.012 (42%) |  |
| Unknown | 496 | 315 | 181 |  | 496 | 286 | 210 |  |
| Phototype (Fitzpatrick classification) |  |  |  | <0.001 |  |  |  | 0.3 |
| low | 1.617 (39%) | 1.013 (42%) | 604 (35%) |  | 1.617 (39%) | 626 (40%) | 991 (39%) |  |
| medium | 2.503 (61%) | 1.380 (58%) | 1.123 (65%) |  | 2.503 (61%) | 925 (60%) | 1.578 (61%) |  |
| high | 0 (0%) | 0 (0%) | 0 (0%) |  | 0 (0%) | 0 (0%) | 0 (0%) |  |
| Unknown | 79 | 66 | 13 |  | 79 | 30 | 49 |  |
| Mutation of MC1R gene |  |  |  | <0.001 |  |  |  | <0.001 |
| MC1R=0 | 1.098 (27%) | 524 (22%) | 574 (34%) |  | 1.098 (27%) | 295 (19%) | 803 (31%) |  |
| MC1R≠0 | 3.040 (73%) | 1.903 (78%) | 1.137 (66%) |  | 3.040 (73%) | 1.257 (81%) | 1.783 (69%) |  |
| Unknown | 61 | 32 | 29 |  | 61 | 29 | 32 |  |
| Gender |  |  |  | 0.7 |  |  |  | <0.001 |
| male | 1.861 (44%) | 1.096 (45%) | 765 (44%) |  | 1.861 (44%) | 760 (48%) | 1.101 (42%) |  |
| female | 2.338 (56%) | 1.363 (55%) | 975 (56%) |  | 2.338 (56%) | 821 (52%) | 1.517 (58%) |  |
| Family history of melanoma |  |  |  | 0.061 |  |  |  | 0.022 |
| none | 3.537 (92%) | 2.152 (91%) | 1.385 (93%) |  | 3.537 (92%) | 1.381 (91%) | 2.156 (93%) |  |
| yes | 311 (8.1%) | 206 (8.7%) | 105 (7.0%) |  | 311 (8.1%) | 142 (9.3%) | 169 (7.3%) |  |
| Unknown | 351 | 101 | 250 |  | 351 | 58 | 293 |  |

*Continuous variables are described by using the median and the interquartile range (IQR), while the categorical variables are summarized through the absolute frequencies and the column percentages*

** Fisher's test and Pearson's Chi-squared test for categorical variables, and Wilcoxon rank sum test for quantitative variables*

**Table S3.** Common nevi effect on melanoma risk in Young individuals (A) and Elderly individuals (B): raw and adjusted odds ratios (OR)*

**A**

| **Study** | **Median (naevi)** | **Raw OR** | **Adjusted OR** | **Gender** | **atypical** | **hair1** | **faireye** | **photo2** | **mut (MC1R)** |
| --- | --- | --- | --- | --- | --- | --- | --- | --- | --- |
| **Gruis, 2001** | 36 | 1.50 | 1.50 |  |  |  |  |  |  |
| **Ribas, 2007** | 12 | 0.82 | 0.89 |  |  |  |  |  | x |
| **Fargnoli, 2008** | 30 | 10 | 6.46 |  | x |  |  |  |  |
| **Bishop, 2009** | 20 | 5.52 | 5.20 |  |  |  | x |  |  |
| **Stratigos, 2009** | 5 | 4 | 1.55 |  | x |  |  |  |  |
| **Ghiorzo, 2012** | 17.5 | 2.33 | 1.58 |  | x |  |  |  |  |
| **Polsky, 2014** | 45 | 3.26 | 3.29 |  |  | x |  |  | x |
| **Guida, 2015** | 25 | 2.40 | 3.49 |  |  |  | x |  |  |
| **TOTAL** |  |  | **2.56** |  |  |  |  |  |  |
| **Menin, 2011** | 30 | 1.35 | 0.29 | x | x |  |  |  |  |
| **Puig, 2013** | 75 | 0.71 | 0.33 | x |  |  |  | x |  |
| **TOTAL** |  |  | **1.68** |  |  |  |  |  |  |

**B**

| **Study** | **Median (naevi)** | **Raw OR** | **Adjusted OR** | **Gender** | **atypical** | **faireye** | **photo2** | **mut (MC1R)** | **interm** | **Cont_ Sunexpo** | **Fam2** |
| --- | --- | --- | --- | --- | --- | --- | --- | --- | --- | --- | --- |
| **Gruis, 2001** | 6 | 6.15 | 6.35 |  |  |  |  | x |  |  |  |
| **Ribas, 2007** | 12 | 0.80 | 1.04 | x |  |  |  |  |  |  |  |
| **Fargnoli, 2008** | 5 | 1.66 | 1.42 |  |  |  | x |  |  |  |  |
| **Bishop, 2009** | 12 | 2.65 | 2.43 |  | x |  | x |  |  |  | x |
| **Stratigos, 2009** | 5 | 4.33 | 8.58 |  |  | x |  |  |  |  |  |
| **Ghiorzo, 2012** | 5 | 0.65 | 0.70 |  |  | x |  |  |  | x |  |
| **Guida, 2015** | 25 | 3.71 | 4.81 |  |  | x |  |  | x |  |  |
| **TOTAL** |  |  | **2.06** |  |  |  |  |  |  |  |  |
| **Menin, 2011** | 5 | 1.64 | 1.64 |  |  |  |  |  |  |  |  |
| **TOTAL** |  |  | **2.00** |  |  |  |  |  |  |  |  |

** Adjustment variables applied to each study for young (A) and elderly (B) people, with median values, raw ORs and adjusted ORs
For the adjustment, we considered only the most clinically and statistically significant variables, such as intermittent sun exposure, continuous sun exposure, skin type (based on Fitzpatrick classification or tanning ability), hair and eyes colour, family history of melanoma and mutation of MC1R gene.
These variables are classified as follows:*

*-* $interm=\left\{ \begin{aligned} 0 if intermittent sun exposure\leq3 hours \\ 1 if intermittent sun exposure>3 hours \end{aligned} \right.$

*-* $cont\_Sunexpo=\left\{ \begin{aligned} 0 if continuous sun exposure=0 hours \\ 1 if continuous sun exposure>0 hours \end{aligned} \right.$

*-* $photo2=\left\{ \begin{aligned} 1 if skin type=I or II \left( low \right) \\ 2 if skin type=III or IV \left( medium \right) \\ 3 if skin type=V or VI \left( high \right) \end{aligned} \right.$ *-* $hair1=\left\{ \begin{aligned} 0 if hair=red \\ 1 if hair=blonde \\ 2 if hair=dark \end{aligned} \right.$ *-* $faireye=\left\{ \begin{aligned} 0 if eye=light (blue/green/grey) \\ 1 if eye=brown/black \end{aligned} \right.$ *-* $fam2=\left\{ \begin{aligned} 0 if family history=none or no melanoma \\ 1 if family history of melanoma=yes \end{aligned} \right.$ *-* $mut=\left\{ \begin{aligned} 0 if MC1R=wild type \\ 1 if MC1R=one or more variants \end{aligned} \right.$

**Figure S1.** Forest plot of adjusted ORs for the effect of common nevi on melanoma risk in the older age group, without Ghiorzo’s study

**Figure S2.** Forest plot of adjusted ORs for the effect of common nevi on melanoma risk in the younger age group, excluding studies by Stratigos and Ribas

**Figure S3.** Forest plot of adjusted ORs for the effect of common nevi on melanoma risk in the older age group, excluding studies by Stratigos and Ribas

**Figure S4.** Forest plot of adjusted ORs for the effect of common nevi on melanoma risk in the younger age group, with the inclusion of studies by Menin and Puig

**Figure S5.** Forest plot of adjusted ORs for the effect of common nevi on melanoma risk in the older age group, with the inclusion of studies by Menin and Puig

**Table S4.** Atypical nevi effect on melanoma risk in Young individuals (A) and Elderly individuals (B)

**A**

| **Study** | **Raw OR** | **Adjusted OR** | **Gender** | **common** | **hair1** | **faireye** | **photo2** | **interm** | **Fam2** |
| --- | --- | --- | --- | --- | --- | --- | --- | --- | --- |
| **Fargnoli, 2008** | 7.34 | 5.50 |  |  |  |  | x |  |  |
| **Bishop, 2009** | 2.54 | 1.58 |  | x |  | x |  |  |  |
| **Stratigos, 2009** | 14.59 | 20.68 |  |  |  |  |  | x |  |
| **Kanetsky, 2010** | 8.18 | 8.29 | x |  | x |  |  |  |  |
| **Ghiorzo, 2012** | 4.31 | 6.93 |  |  |  |  |  | x |  |
| **Guida, 2015** | 1.36 | 1.36 |  |  |  |  |  |  |  |
| **TOTAL** |  | **4.84** |  |  |  |  |  |  |  |
| **Menin, 2011** | 55.56 | 55.38 | x |  |  |  |  |  |  |
| **Puig, 2013** | 1.77 | 1.18 |  |  |  |  |  |  | x |
| **TOTAL** |  | **5.71** |  |  |  |  |  |  |  |

**B**

| **Study** | **Raw OR** | **Adjusted OR** | **Gender** | **common** | **faireye** | **photo2** | **mut** | **Cont_Sunexpo** | **Fam2** |
| --- | --- | --- | --- | --- | --- | --- | --- | --- | --- |
| **Gruis, 2001** | 1.20 | 0.68 |  | x |  |  | x |  |  |
| **Fargnoli, 2008** | 1.09 | 1.19 |  |  |  | x |  |  |  |
| **Bishop, 2009** | 2.54 | 2.02 |  | x |  |  |  |  | x |
| **Kanetsky, 2010** | 3.61 | 3.08 | x |  |  |  |  |  |  |
| **Ghiorzo, 2012** | 0.93 | 0.93 | x |  | x |  |  | x |  |
| **Guida, 2015** | 2.47 | 3.54 |  |  | x |  |  |  |  |
| **TOTAL** |  | **1.71** |  |  |  |  |  |  |  |

**Figure S6.** Forest plot of adjusted ORs for the effect of atypical nevi on melanoma risk in the younger age group, without Bishop’s study

**Figure S7.** Forest plot of adjusted ORs for the effect of atypical nevi on melanoma risk in the younger age group, with the inclusion of studies by Menin and Puig
